# A Multidisciplinary Approach to Research in Small-Scale Societies: Studying Emotions and Facial Expressions in the Field

**DOI:** 10.3389/fpsyg.2016.01073

**Published:** 2016-07-18

**Authors:** Carlos Crivelli, Sergio Jarillo, Alan J. Fridlund

**Affiliations:** ^1^Facultad de Psicología, Universidad Autónoma de MadridMadrid, Spain; ^2^Anthropology Division, American Museum of Natural History, New YorkNY, USA; ^3^Department of Psychological and Brain Sciences, University of California, Santa Barbara, Santa BarbaraCA, USA

**Keywords:** multidisciplinary approach, indigenous societies, methods, facial expressions, emotion, culture

## Abstract

Although cognitive science was multidisciplinary from the start, an under-emphasis on anthropology has left the field with limited research in small scale, indigenous societies. Neglecting the anthropological perspective is risky, given that once-canonical cognitive science findings have often been shown to be artifacts of enculturation rather than cognitive universals. This imbalance has become more problematic as the increased use of Western theory-driven approaches, many of which assume human uniformity (“universality”), confronts the absence of a robust descriptive base that might provide clarifying or even contrary evidence. We highlight the need for remedies to such shortcomings by suggesting a two-fold methodological shift. First, studies conducted in indigenous societies can benefit by relying on multidisciplinary research groups to diminish ethnocentrism and enhance the quality of the data. Second, studies devised for Western societies can readily be adapted to the changing settings encountered in the field. Here, we provide examples, drawn from the areas of emotion and facial expressions, to illustrate potential solutions to recurrent problems in enhancing the quality of data collection, hypothesis testing, and the interpretation of results.

## Introduction

Translating knowledge from one scientific domain to another is always constrained by differences in methods, description, goals, and explanation. Researchers who wish to establish facts about human cognition are also plagued by problems in translation, but they may not realize it. The translation problems are partly due to the fact that *people differ cognitively from culture to culture*. How and how much is uncertain, but much cognitive science research proceeds as if this difference did not matter.

Cognitive science’s foundational goal of multidisciplinary collaboration among disciplines has gradually receded. Nowadays, psychologists produce the majority of the publications, are overrepresented in cognitive science’s conferences, and psychological findings are especially compelling to the media and funding agencies (Gentner, 2010). Psychology’s dominant status within cognitive science has also set the research agenda in the search for shared mechanisms that produce behavior and general laws that predict and govern it. The former approach collided with anthropologists’ findings on diversity across human populations (Shweder, 2012). Moreover, psychologists and anthropologists’ research goals and methods previously seen as complementary now seem incompatible (Bender et al., 2010; Beller et al., 2012).

Our own field, that of emotion and facial expression, is a sprawling area rich in controversies. All in all, the predominance of experimental psychologists searching for general laws, the tradition of conceptualizing emotions as natural entities with casual properties, the use of canonical operationalizations (e.g., theory-driven facial expression matching-to-sample studies), and the generation of emotion theories based on US American samples as the normative population, have left no room for multidisciplinary collaborations with anthropologists (Russell, 1991; Wierzbicka, 2014; Plamper, 2015).

In this paper, we discuss the benefits for an effective integration of anthropology within cognitive science. Using examples from our own research on emotion and facial expression, we highlight challenges that researchers are likely to encounter when they attempt to conduct studies in small-scale, indigenous societies. By considering these issues, we have begun to resolve key questions only because we have relied on the combined strengths of a multidisciplinary research team established by psychologists and anthropologists carrying out their research beyond the necessarily artificial Western laboratory setting.

## Why Is Anthropology So Important for Cognitive Science?

Although it is now more accepted that obtaining human cognitive science data from diverse societies enriches the data and any conclusions drawn from them, the anthropological perspective suggests that, even within any one society, the quality of human cognitive science data can be improved. The main shortcomings identified by a more anthropological perspective are: the use of narrow samples, the inattention to diachronic features of behavior, and inadvertent ethnocentric bias.

### Narrow Sampling

A central issue concerns claims that are often made about uniformity (“universality”) versus diversity in human behavior. How confident can we be regarding these claims? In evaluating any of them, we must remember that sampling from the human population has been largely restricted to Western, educated, and industrialized societies, and mainly college students at that (Arnett, 2008; Henrich et al., 2010)^1^. But the solution to narrow sampling should not be simply to dispatch groups of psychologists to all possible field sites in order to study as many “non-normative” societies as possible. Rather, narrow sampling can be overcome by collaborating with researchers from other disciplines (e.g., anthropologists but also linguists, sociologists or human geographers) with extensive knowledge in the field (Astuti and Bloch, 2010; Majid and Levinson, 2010).

Anthropologists’ training leads them to gravitate toward studying “non-standard populations.” In these less-studied societies, anthropological accounts, among other sources, are used to provide behavioral descriptions, representations of the world, and indigenous frames of reference. Knowledge is conceived not as a fixed, timeless *corpus* of accumulated facts, but as relational, extensive and dynamic, and as such it is always provisional and amenable to updating, re-tests and replications.

### Diachronic Features of Behavior

In anthropology, assaying the spatial dispersion of a human phenomenon (i.e., studying as many diverse indigenous societies as possible) is only half the job. It is also essential to study a phenomenon’s temporal dimensions—its diachronic properties. Changes over time are extremely relevant for avoiding misinterpretations of present behavioral patterns and their functional accounts. The temporal dimension is often given short shrift in psychological approaches, possibly because of psychology’s long history of behavioral explanation via static trait ascriptions in theories of personality. This can lead to a tendency to view cultural constructs as timeless and fixed, instead of as dynamical systems (Schmittmann et al., 2013).

Considering culture dynamically implies that cognitive scientists will often need to consider returning to the field periodically in order to update their cultural databases regularly. Accordingly, advances in techniques such as network analysis or text analysis can be employed for mapping within-culture diachronic changes rigorously and quantitatively (Borgatti et al., 2009; Iliev and Ojalehto, 2015). For example, a recent debate between a group of psychologists studying “emotional” vocalizations among the pastoralists Himba of Namibia and an anthropologist with extensive experience in the same society is illustrative of two different attitudes toward reporting and describing the members of an indigenous society. The psychologists depicted Himba participants as people isolated from other cultural groups (Sauter et al., 2010). In their eagerness to describe their participants as visually “isolated” from other cultural groups living in settlements geographically distant from urban centers, the psychologists did not report that the Himba they sampled had been in contact with other cultural groups since the 1860s (Gewald, 2010). Therefore, the anthropologist’s main criticism was aimed at psychologists’ deficient descriptions and how they overlooked relevant diachronic information.

### Ethnocentrism

The concept of ethnocentrism refers to a bias toward one’s in-group (Gumplowicz, 1879; Sumner, 1906). Accordingly, Western researchers going to the field must realize that the Western cultural frame of reference shapes the way we categorize reality, build our theories, test our hypotheses, and make universal assumptions based on “canonical” English descriptors (Wierzbicka, 2014). To overcome this problem, anthropologists are warned of these risks beforehand. Even well-trained researchers can unknowingly be trapped by their cultural backgrounds, which leads to biased descriptions and explanations of events (DeVita, 1990).

The anthropological method works to limit ethnocentrism by having investigators (i) remain in the field for a sufficient period of time, (ii) understand and speak the vernacular, (iii) conduct participant observation, (iv) discern what are termed the “rich points” within the culture (i.e., those events that we find unexplainable or collide with our beliefs and previous theories), and (v) pursue answers to the research questions posed by such rich points (Agar, 1986, 1996). Consequently, the investigator’s stance during the first stages of fieldwork is agnostic and exploratory.

Subtle forms of ethnocentrism may persist nonetheless, especially when doing fieldwork in indigenous societies. One striking example is the Western conception of the distinction between when we are in private (i.e., in solitude) vs. in public (i.e., in the presence of others). Posed as a dichotomy, this distinction was used by emotion researchers to divide facial behaviors into two classes: those produced alone were genuine “emotional expressions,” whereas those produced in the presence of others were “social” expressions that were subject to faking or masking (Ekman and Friesen, 1969; Ekman, 2003). For anthropologists, the public vs. private dichotomy can be arbitrary and unproductive (see, for example, Good and Chanoff, 1991). Moreover, the conception of the private, inner self, rarely if ever revealed in public, seems to be distinctly Western, with some historians tracing it to St. Augustine (Cary, 2000).

Indeed, the situations in which a Trobriand Islander is completely alone are rare, and they are mainly associated with the practice of some types of magic (*megwa*)^2^. In these situations, magic practitioners confine themselves at night in the bush or in solitary cliffs, but they are far from alone. Magic spells differ, but most have in common the recalling of deceased matriclan ancestors to help them in their magic (Jarillo de la Torre, 2013; Mosko, 2014). Thus when these sorcerers are physically alone, psychologically speaking, there is always an implicit audience to whom they speak and with whom they interact (Fridlund, 1991, 1994).

### The Problem of Description

In the 18th century, slow but systematic work by taxonomists created solid ground for generating theories in biology. In the same vein, early animal behaviorists stressed the importance of pre-theoretical description, and advocated segmenting complex behavior sequences into smaller units for labeling and classification (Hinde, 1970). There may be subjectivity in how any stream of behavior is parsed, but such efforts have an objective content and application (Blurton-Jones and Woodson, 1979). Anthropologists’ training is a special strength to the field due to its focus on context as well as the use of robust descriptive strategies. Moreover, native anthropologists are able to grasp significant information from observing a given context, making accurate inferences based on objects, body decorations, or even the ecological environment (Young, 1998; Senft and Basso, 2009; Medin and Bang, 2014).

In the field of emotion, the research agenda has largely been one of theory validation over empirical approaches (Kagan, 2007). Accordingly, psychologists have relied for description mostly on their own individual and cultural frames of reference, assuming that those frames were normative and not culturally constrained. In fact, Rai and Fiske (2010, p. 107) stated that, “psychological theory over the past 40 years has been formulated mostly on the basis of prior theory, data, and intuitions.” Neglecting bottom–up strategies has led researchers to build theories on problematic assumptions (Fernández-Dols, 2006), especially when applicable and robust bottom–up methods have been well-honed in other disciplines (Jack et al., 2012). We next illustrate how Western preconceptions may have hampered efforts at accurate description and explanation.

## Rethinking Cross-Cultural Comparisons

One prominent and pervasive uniformitarian cross-cultural approach assumes that basic underlying psychological processes are universal, whereas culture just taints with minimal variation the uniform products of those universal processes in the resulting phenotype (Berry et al., 2002). When applied to emotion research, this uniformitarian approach tests hypotheses originated by Western theories within a given cultural tradition (e.g., romantic love). Usually, the studies are conducted in a Western-style laboratory, the participants are Westernized, educated, middle-class college students (who watch U.S. and European TV, listen to American music, and whose course readings are often just translated U.S. texts), and the data are leveraged to verify or falsify Western-centric hypotheses on cultural differences.

Before going to the field, researchers following the uniformitarian approach typically discuss and establish the study design—a replication of the Western-society study. In some cases, researchers do not go to the field, but use collaborators who follow instructions pre-established in the Western laboratory (Ekman, 1972, p. 281; Tracy and Robins, 2008, p. 520). Once in the field, the instructions are translated to the local languages. Translations may be most vulnerable to Western bias, given that direct translations are sometimes not found. Inexact substitutes must be arranged on the spot, and even with fairly exact translations, unappreciated nuances and idioms in both source and destination vernaculars can compromise the validity of the forward translation of the tasks and the back translations of the responses. Researchers frequently rely on local translators (Ekman, 1972; Sorenson, 1976; Sauter et al., 2010; Gendron et al., 2014a,b); they may be differentially skilled at forward and backward translation. At the end of the process, the data gathered in the field are used as a comparison group for the Western “normative” data.

Older views of cognition, which considered the contents of thought to be local and culture-bound, whereas the modes of cognitive processing were universal and culture-free, are now considered untenable (Park and Huang, 2010; Kitayama and Uskul, 2011; Beller and Bender, 2015; Bender and Beller, 2016). Culture affects both cognitive content and processes, a fact that makes a reified view of “culture,” used as a mere nominal variable in prior uniformitarian approaches, woefully inadequate to the task of presenting how pervasively culture affects how people differ in the ways they experience, discriminate, and categorize their worlds (Beller et al., 2014; Ojalehto and Medin, 2015).

Historically, the content-process distinction prompted the isolation of anthropologists from psychologists’ research agendas. Whereas psychologists were focused on cognitive processes under the assumption that they were universal at individual and cultural levels (Bender et al., 2010; Beller et al., 2012), anthropological findings were footnoted as minor variants or demoted to the status of anecdotal evidence (e.g., Ekman, 1980). Re-establishing collaborations with anthropologists is wholly justified by the amount of evidence they have gathered on content variation, the generation of hypothesis and the enhancement of external validity, their expertise on overcoming the challenges of the home-field disadvantage, and, specially, the importance of integrating different but complementary methodological approaches within cognitive science (Medin et al., 2010; Bender and Beller, 2011; Bender et al., 2012; Kitayama, 2012)^3^.

## The Integration of Psychological and Anthropological Methods

We propose the use of mixed methods research as a way to take advantage of dual expertise: that of psychologists in quantitative techniques, and social anthropologists in qualitative methods (Tashakkori and Teddlie, 2010; Creswell, 2014). This integration occurs in the field, where the anthropologist and the psychologist guide each other and produce a true cross-disciplinary collaboration between two different research traditions (Agar, 2013). Next, we will discuss the implications for future research when integrating psychological and anthropological methods in the field. We will focus on the exploratory, testing, and control phases.

### The Exploratory Phase

In the exploratory phase, data are obtained initially by qualitative methods in order to aid the development of research instruments, which then lead to the design and testing of hypotheses using quantitative methods (Greene et al., 1989). This is in stark contrast to previous universalist approaches, in which researchers are dispatched to diverse cultures with measures already in hand, a strategy that is highly prone to the pitfall of “beating down” the phenomenon to fit the study.

Indeed, the initial exploratory period we consider mandatory may be especially revealing, in several ways. First, previous results reported for that society may prove to be dated, or else to have been misinterpretations or misunderstandings due to incomplete knowledge of the cultural context, or no longer indicative of current practices in the culture, and these findings may require update, correction, or recontextualization. Second, more complex designs may emerge which mandate further qualitative data (e.g., interviews), or the use of multiple methods in a convergent design after the exploratory phase (Creswell et al., 2003). For example, investigators may travel to diverse societies hoping to study the emotional lives of their members, Likert scales in hand, only to find that these members may find it off-putting to be asked openly about their “feelings” and emotions.

Thus, the exploratory phase—taking place before the study design and data collection—is crucial for enhancing the validity of the study, the quality of the data, and the robustness of interpretation. Qualitative anthropological research demands “long-term and open-ended commitment, generous attentiveness, relational depth and sensitivity to context” (Ingold, 2014, p. 384)^4^. To achieve these, participant observation, speaking the vernacular, and building rapport are the three facets in the exploratory phase that need to be accomplished in order to proceed to the design and testing phase. We survey each of these components.

#### Participant Observation

The best way to learn what is implicit and explicit in another culture is through participant observation. Participant observation can best be defined as the active process by which the researcher strives to understand the socio-cultural universe of the host community while participating in the enactment of that universe. In that sense, the first step is to engage in the indigenous society’s daily activities and to live by the standards of the host community. Participant observation is a long-term investment. The more time and resources investigators expend doing participant observation, the better the quality and interpretation of data they will obtain (Dewalt et al., 1998). Anthropologists have discussed the extent to which emotional involvement and degree of participation can and should determine the outcomes (Clifford and Marcus, 1986). Indeed, moderate participation (e.g., rarely interacting in daily life, not living with an adopted family) provides poor insights when compared with active and complete participation (Spradley, 1980).

Active participant observation yields many benefits. First, constant presence in daily events and regular interaction with locals offers an explicit gesture of commitment toward local social obligations, diminishing the hosts’ reluctance to communicate explicit knowledge and enhancing the researcher’s implicit knowledge of the indigenous society’s culture. Second, the researcher gains access to all sorts of natural behaviors in a variety of situations and to implicit knowledge prior to linguistic confirmation (Bloch, 1991). Third, informal encounters in daily life function as rich exchanges of information in which the researcher builds a network of relations who voluntarily share information and provide insights regarding diverse aspects of the culture. Fourth, behaviors not directed to the participant observer can be accessed by observing and listening others while they interact.

In our own area of research, active participant observation provided us insight into a category of behavior that would be “irrelevant” by Western standards but meaningful to Trobrianders. When Trobriand children and adolescents were asked to match emotion labels to prototypical facial expressions of “emotion”, the modal category was the gasping “fear” face (Crivelli et al., 2016). In successive interviews, Trobrianders described the gasping “fear” face using a naïve behavioral descriptor: *ekapunipuni matala* (literally, his/her eyes are wide open). By following these unexpected “rich points” (Agar, 1986), we discovered that in the Trobriand Islands, people associate wide-open eyes either with anger (*leya*) or supernatural spiritual beings.

In fact, when adults want to instill “fear” in children, they imitate what is said to be the visage of evil spirits with wide-open eyes. When the sender wants others to freeze or flee, the person displays an *ekapunipuni matala* face (i.e., a gasping face). This is culturally sanctioned in a Trobriand foundation myth (*liliu*) that explains the origins of ancestral spirits. Baloma, a recently deceased woman, returns to her village to care for her daughter’s newborn, as is customary in the Trobriand Islands. When her daughter’s husband spots her big wide-open eyes (*matala mabulubolela*) in a dark corner of the house, he becomes frightened and spills soup on her, offending her and leading her to invoke a curse that henceforth human beings will not be able to see the spirits of the dead (who take their name from the woman in the myth and are now known as *baloma*; see Malinowski, 1916; Jarillo de la Torre, 2016)^5^. The *baloma* spirits, like other supernatural agents in Trobriand cosmology (i.e., *kosi*, ghosts; *itona*, evil spirits; *yoyowa*, flying witches), are described as having unnaturally large, wide-open eyes.

**FIGURE 1:**
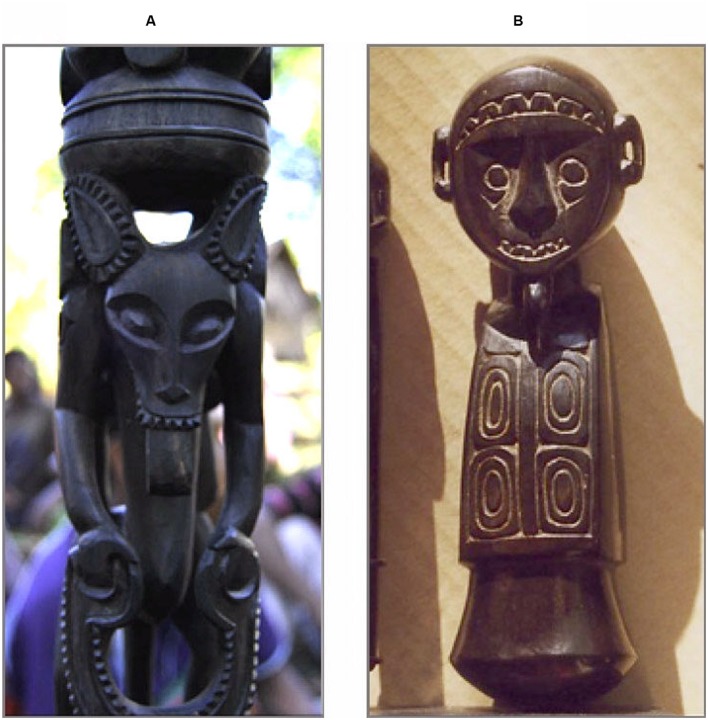
**The *Ekapunipuni Matala*, or the wide-eyed *gasping* face.** **(A)** Walking stick (*kaitukwa*) from the Trobriand Islands carved to represent a flying witch (*yoyowa*), with big glowing eyes and a gaping mouth to swallow people (photo: Sergio Jarillo); **(B)** Betel nut mortar (*kaimili*), Trobriand Islands (former Harry Beran Collection, HB578 - published in Beran, 1980, p. 23, item 39. Photo: Radomir Joura).

This kind of complexity is readily explained in terms of a behavioral-ecology view of facial displays; the receiver of the *ekapunipuni matala* signal sees a supposedly “fearful” face in the sender, but rather reacts to it as a signal of an agonistic encounter in which one must either freeze or flee (Krebs and Dawkins, 1984; Fridlund, 1994). We also realized that the production of the *ekapunipuni matala* face has become iconic in Trobriander’s material culture. *Yoyowa*—flying witches—and other supernatural beings are represented in carvings (**Figure 1**) as having big glowing eyes and jaws that are dislocated to swallow people (Jarillo de la Torre, 2013; Aldridge and Beran, 2014).

#### Advantages of Speaking the Vernacular

Speaking the local language while doing research in indigenous societies carries two advantages. First, it indicates the researcher’s commitment to integrate with and acculturate to the locals’ view, which builds rapport and maximizes the ability to obtain relevant data (Newman and Ratliff, 2001). Second, it reduces the problems inherent in translation (Naroll and Cohen, 1970).

Learning the vernacular also shows respect for the locals’ socio-cultural values (Everett, 2001). The people in local communities are usually pleased with the humble approach of taking the time and effort to learn their language (Duranti, 1997). As this happens, interactions become more like informal interviews in which relevant chunks of information can be extracted. All actors in those encounters are valuable informants and all the interactions serve not only to validate information previously gathered, but also to prepare the terrain for future assessments. As researchers begin to form a network of relationships, they can ascertain whether a given person’s information is reliable or not (e.g., sometimes very accessible bilingual informants tend to “embellish” facts with confabulated stories). Moreover, the researcher can start having local intuition about certain domains.

Using local interpreters may appear to be an appealing alternative, but engenders several undesirable consequences. Interpreters typically: (i) select the sample of participants or suggest which participants to choose, (ii) influence procedures while translating instructions out of their eagerness to assist in the research, and (iii) tend to interpret results when translating responses, thus inadvertently switching from direct to indirect translations (Phillips, 1960). A factor that looms over all use of translators is the issue of their intermediation and its effect on the relationships between researchers and participants. Most translators act as gatekeepers of, or brokers to, the host community (Obstfeld et al., 2014). This *de facto* monopoly on access to local information can hide local loyalties and enmities, rewards, and stigma, and these factors can lead participants to behave and respond in certain ways that remain unknown to the researchers (Borchgrevink, 2003). Speaking the vernacular allows researchers to build their own networks of relations within the host community, and thereby become central actors themselves, with some autonomy and independent access to information.

Field researchers should avail themselves of local commentators or research partners. Whereas translators fulfill a “mechanical” duty (e.g., the more or less literal translation of a series of instructions), local collaborators can discuss, in accordance with researchers who already know the vernacular, the appropriateness of a task in obtaining relevant information.

#### Building Rapport

In most indigenous societies, many of the situations that a Western participant would find reasonable (e.g., strangers asking for our “feelings”) are treated with suspicion (for a similar point, see Sorenson, 1976, p. 140). Without having established rapport with the host community and observing how its members naturally interact, researchers will find it very difficult to get useful responses to topics like anger, social conventions, stigmas, and taboos. As an example, the first co-author of this paper arrived at the premature conclusion that young adults in central Kiriwina (Trobriand Islands) were not sufficiently abstract to engage in an interview on emotion concepts; when asked, they either remained silent or just provided short answers. Indeed, elders confided to him that the youngsters did not know the myths and customs of their people, and that we should not waste our time and gift them with betel nuts. Following the elders’ advice, the first co-author would have gathered data provided by a very specific Trobriand subsample, underestimating diversity. Similarly, ethnobiologists mainly rely on male elders as their source of information, generating gender-imbalanced fieldwork (Pfeiffer and Butz, 2005).

The second co-author of this paper, however, an anthropologist with more than 2 years of experience in that field site and knowledge of the customs and vernacular, knew otherwise. As he explained, although the young adults in central Kiriwina were warming to the psychologist’s questions on emotions, they did not feel sufficiently confident to reveal their thoughts on such personal issues. This was a problem of trust and rapport for the Trobrianders, who learn to be wary about to whom and for what purpose they disclose personal information; such information can be used by others to benefit or harm their reputations. In this society, individuals who spend sufficient time in participant observation and rapport-building will receive very valuable information on a full range of topics (e.g., for incest and morality, see Astuti and Bloch, 2015).

As another example, the regular interaction with Trobriand children and adolescents provided valuable insights on the disparate uses of indigenous terms for shame and embarrassment compared to older and educated locals (for further discussion on this issue, see Fernández-Dols and Crivelli, 2014). This fact is relevant because anthropologists tend to build networks of informants with specific sets of features that conform to the scope of their research (e.g., they will seek out people with esoteric knowledge if they are studying a culture’s secret rituals), but they rarely consider children and adolescents’ reports worth the effort.

### The Testing Phase: Cautionary Notes on Designing in the Field

**FIGURE 2:**
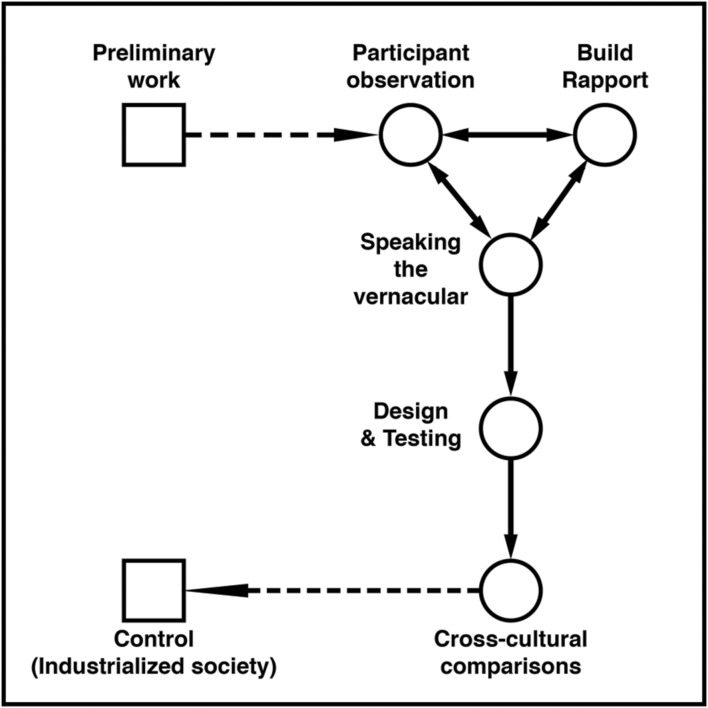
**A framework for conducting studies in indigenous societies with a multidisciplinary research group made up of anthropologists and psychologists.** Squares denote the research path characteristic of a Western laboratory setting; circles denote the research path typical of fieldwork in an indigenous society.

Much social science research has been directed toward testing hypotheses derived from formal theories. One alternative that reduces ethnocentrism is the development of theories grounded in empirical data culled from socio-cultural interactions. Anthropology offers an excellent strategy for discovering grounded theory (Glaser and Strauss, 1967). The use of qualitative methods in the exploratory phase is followed by the design of studies and interventions to test the relevant hypotheses (**Figure 2**).

Although we agree that a general research agenda and set of research questions should be set prior to fieldwork, the specific hypotheses to be tested and the design of studies to test them should follow, and depend upon, the exploratory phase (Lopez et al., 1997). As we suggested earlier, importing to host cultures “standard” procedures and task instructions suitable for Western undergraduate participants is typically unproductive (Medin and Atran, 2004). Once tests are developed that are honed to the host culture (e.g., sorting tasks with meaningful objects within that indigenous society), they are much easily exported back, *mutatis mutandis*, to Western society participants. In any case, researchers can find situations in which exporting tests and tasks to the Western society can be extremely challenging. For example, all Trobrianders know what *mwasila* (magic of attraction, radiance) means or the consequences derived from carrying it. Trobrianders associate to *mwasila* the capacity to influence the minds of those who come into contact with it, some sort of raptured enchantment. Thus, the classic two-culture comparison will be problematic because the concept of “mwasila” is alien to participants from Western industrialized societies.

The need for a prior exploratory phase is provided by another example, which we encountered in two different field sites. The issue relates to psychological scaling. Earlier, we suggested that, for some cultures, using self-reports would be seen as strange or preposterous. And certainly, any scaling has to be tested in advanced. The vernacular provides hints on counting systems (Bender and Beller, 2008), but daily interaction confirms what level of measurement should be used when designing quantitative studies in the field (Bloch, 1991). We found that data gathered via self-report with Trobrianders could use scales, but they had to be restricted to a maximum of 4-point ordinal unipolar scales; e.g., *gala* (nothing), *pikekita* (a little), *sena* (a lot, very), and *komwedona* (all, everything). Likewise, Mwani communities in Cabo Delgado Province (Mozambique) will not go beyond the same basic ordinal scale. Thus, for these societies, the standard practice in cross-cultural psychology of using 7- or 9-point Likert scales for self-report surveys and questionnaires is likely to confuse many participants and produce erroneous, unreliable data. There is no substitute for designing and scaling in the field after acquiring familiarity with the language and culture.

### The Control Phase: Flipping What is Normative

A potential solution to overcome the home-field disadvantage—especially suited when psychologists and anthropologists collaborate—is to use the indigenous society as the starting point (Medin et al., 2010). In this approach, we “flip” the usual etic tack and acknowledge that, as “experts” in our own Western society’s shared meanings, it is typically easier to work out the experimental conditions and measures in the field and bring them home to our Western laboratories than the other way around. We concede that it is more challenging to use the indigenous society as our normative benchmark, but the benefits of the exploratory phase aided by anthropological expertise yields indispensable benefits.

Most researchers not interested in developmental studies will gather data in Western labs by sampling from a gender-balanced group of young adults. Accordingly, the Western sample will be matched by a similar sample in the field. But once in the field, the researcher is vulnerable to many unexpected difficulties in order to match samples. For example, women could be inaccessible (especially for male researchers); and adults engaged during the day in subsistence activities such as fishing or gardening could make it unfeasible to test any hypothesis that requires daylight (e.g., sorting tasks, matching stimuli to emotion labels). Moreover, researchers have to consider that diversity within a society should be expected. It is notable that, among some indigenous societies that lack mass media, the members who are least exposed to Western influence may be their children and adolescents, yet studies of these populations are scarce (see Ekman, 1972; Pica et al., 2004).

Several factors make working with children and adolescents difficult nonetheless. First, rapport can be difficult to establish, and younger people are unlikely to share information unless the researcher has established trust and rapport and mastered the vernacular. Second, attaining young cross-cultural samples that are age-equivalent can be unrealistic because many indigenous groups lack written records of birthdates. Moreover, members of such groups often figure time according to harvest seasons and lunar cycles, and they have neither written nor oral conventions by which to demarcate finer gradations of time. Third, particular aspects of child and adolescent development in certain societies may make only certain subsamples of those indigenous societies comparable to their Western counterparts (Medin and Atran, 2004). For example, young male Mwani adults living in Matemo Island start sailing within the Quirimbas archipelago and the African mainland (*ndima*) before age 20, whereas young female Mwani adults rarely leave their island. For that reason, individuals drawn from female Mwani young adults would be closer to traditional Mwani culture due to their more limited contact with Westerners.

## Putting Theory in Practice: A Trobriand Case

Our defense of a multidisciplinary approach as discussed in this paper is not entirely new. In fact, relevant collaborations between psychologists and anthropologists have already produced important theoretical advances in topics that range from folk biological taxonomies to theory of mind (Astuti, 2007; Atran and Medin, 2008; Medin and Atran, 2008; Wassmann et al., 2013; Beller and Bender, 2015). Moreover, some researchers have considered as a prior step to study emotions in the field accessing the ethnopsychological model of the target population, focusing on the pragmatic rather than on the referential functions of emotion language (Lutz, 1988a,b; Senft, 2009).

We agree with Astuti and Bloch (2012) when they claim that the collaboration between psychologists and anthropologists should not be restricted to using the anthropologist as merely a research assistant with access to an exotic sample in order to export and adapt Western, experimental tasks. Likewise, we also agree with Levinson’s (2012) criticism that it borders on psychological malpractice to gather data from exotic samples located near tourist areas, relying on professional translators, and using inappropriate stimuli and procedures. Indeed, we have seen some examples of dubious multidisciplinary collaboration of psychologists and anthropologists in the field of emotion and facial expression.

One of the most relevant theories of emotion of the last 50 years—Basic Emotion Theory (BET, Ekman, 1972, 2003)—traces its foundational studies to cross-cultural comparisons drawn between Western and Eastern industrialized societies and the hunter-gatherer Fore of the Eastern Highlands (Papua New Guinea). Paul Ekman and Wallace Friesen (psychologists), and Richard Sorenson (anthropologist) went to the field in order to test whether people from a “Stone-Age” culture, visually isolated from the West, were able to judge the same emotion when viewing the same facial expression as Westerners did (Ekman, 1972, 1973, 1980). It is important to note that neither of them spoke the vernacular, nor had they spent a significant amount of time among the Fore. Interestingly, several years after the first expedition published in *Science* (Ekman et al., 1969), the anthropologist criticized the psychologists’ approach in the field (Sorenson, 1975, 1976). Sorenson carefully explained a series of method artifacts that could have overestimated the sounded the uniformitarian results reported in several high impact journals (Ekman et al., 1969; Ekman and Friesen, 1971). Indeed, after spending more time in the field, learning the vernacular and the customs, Sorenson understood what failed in the past and provided some new directions on how to test participants. Sadly, the psychologists did not accept any of the critical points raised by Sorenson and accused him of lying. Ekman argued that Sorenson was “just a cinematographer, not a trained social scientist” (Ekman, 1999, p. 310).

Unlike the previous unsuccessful “multidisciplinary” research project, we have followed a different path. We admit that our proposal can be discouraging for many researchers. Nowadays, young researchers must generate numerous publications in order to enter academia, whereas established researchers, must maintain funding by pursuing safe, programmatic research dictated by ongoing funding initiatives. Multidisciplinary collaborations are long-term investments with uncertain payoffs. Likewise, setting a field site in order to conduct studies in small-scale, indigenous societies is not as simple as building a permanent facility in any “isolated” village in order to test villagers (Astuti and Bloch, 2010). To build a truly multidisciplinary research team, the psychologist should learn the vernacular, conduct participant observation and build rapport with the host community aided and informally trained by the anthropologist. Consequently, the cultural anthropologist should also learn to integrate the strengths of powerful descriptive methods into the generation and testing of hypotheses relying on quantitative methods. The cultural anthropologist and psychologist will read and discuss new topics, new epistemological traditions, research practices, and methods. As referred before, this process of mutual socialization into other’s discipline is time consuming, but over the long-term the benefits greatly exceed the costs.

### The Case of Surprise

We referred earlier to our own area of emotion and facial expression as typifying one in which theory derived on Western presumptions was force-fit into cross-cultural investigations. Specifically, according to Ekman’s BET, surprise was stipulated to be a “basic emotion”: it was phylogenetic, had a restricted range of evolved elicitors (sudden, unexpected events), and produced a prewired, prototypical, pan-cultural facial (i.e., brows raised and opened mouth) and vocal (i.e., a sharp inhalation) expression (Ekman, 2003; Sauter et al., 2010). Here, theory generalized specific observations mainly of Westerners to an uniformitarian claim about human emotion and behavior.

Unfortunately, later experimentation suggested that both the observations and the generalizations about “surprise” were faulty. In defiance of the theory, the relationship between feeling surprised and the production of its predicted facial prototype is very weak (Reisenzein et al., 2006, 2013), and does not generalize to an African indigenous society (Gendron et al., 2014a). How could this error have been avoided? Pre-theoretical descriptions are a better starting point for developing hypotheses than proclamatory uniformitarian theory, and they are crucial for providing alternative hypotheses to existing theories (Fernández-Dols, 2006; Crivelli et al., 2015).

Such descriptions also allow unexpected discoveries. Investigators cannot know what they have not yet seen, and should not arrive at field sites full of preconceptions that may cloud their observations. For example, we unexpectedly found an indigenous signal of surprise in the Trobriand Islands. It is highly stereotyped and frequent in its production and recognition by Trobrianders, but not Westerners. Trobrianders, when surprised, produce neither sharp inhalations nor any typical facial behavior. They hit their tongues against the palate, thereby producing a fast and repeated palatal clicking sound. Additionally, the variety of metaphoric references to the body (e.g., *lopola*—her/his insides) or mind (e.g., *nano-*) provides a rich conceptual system of emotion categories, challenging the traditional notion of a one-to-one correspondence when forcing local translators to fit into a Western (English-based) emotion framework (Senft, 1998; Enfield and Wierzbicka, 2002).

Are there Trobriand terms for our Western “surprise”? *Eyowa lopola* (literally, his or her insides have jumped) could be translated as surprise, but its features (e.g., neutral quality of the feeling, fast timing, closing of the eyes and wincing) better resemble startle (Ekman et al., 1985). Other Trobriand expressions also connote something akin to surprise, but are different in nuance. *Ekau nanogu* (i.e., it has taken my mind, it has raptured my ideas) can be said of any positive surprise and it could be translated as “I am speechless.” This is often conveyed through the aforementioned palatal clicking of the tongue. Other related metaphors include *ekubui lopola* (literally, his or her insides are trembling; meaning he/she is apprehensive) and *kwami lopola*, (i.e., his or her belly has been given a pleasant surprise; meaning the person is pleased). These show how subtleties can underlie what is often reduced to a single Western emotion term (Majid, 2012; Wierzbicka, 2014)^6^.

Moreover, a closer look at the features of *eyowa lopola* illustrates how investigators going to the field should be wary not only of one-to-one translations but also of the reliance on information gathered solely from local translators. Trobrianders typically describe feeling *eyowa lopola* when being called unexpectedly by somebody during a walk at night. Trobrianders will not describe feeling fear or enjoyment until they discern whether the voice from the dark is from a person they know or a stranger (including sorcerers or evil spirits). At first, *eyowa lopola* will be used to describe how they felt when facing an unexpected event. Although these descriptions of feeling *eyowa lopola* match similar eliciting scenarios in the canonical Western script (Ekman and Cordaro, 2011), the facial display that accompanies *eyowa lopola* does not match the script.

As we have seen, the utility of building a descriptive base prior to testing theory-driven hypotheses should be obvious. Exporting preconceived theory to test in the field places blinders on the investigators and constrains the participants to react only within the confines of the theory. Had we ventured to the Trobriand Islands just to test a hypothesis about surprise displays, we would have left only with a confirmation or disconfirmation of our hypothesis, and we would have learned nothing about how Trobrianders experience surprise, categorize types of surprise, and react to each other when they are surprised.

## Conclusion

The shift toward research involving indigenous and remote societies, particularly involving controversial areas like facial expressions and emotion, is welcome but must proceed with care and subtlety. We have addressed the benefits of multidisciplinary research teams with anthropological expertise. We adhere to the proposals made within cognitive science in the pursuit of integrating diversity of practices, epistemologies, and peoples for a better understanding of scientific enquiries (Bender and Beller, 2011; Medin and Bang, 2014). In this paper we have advocated for leveraging the strengths that anthropologists’ methods and expertise can provide when psychologists are interested in testing hypotheses in indigenous and remote societies. Following our past experience in the field of emotion and facial expression, we have illustrated some recurrent challenges that researchers may face in the field and ways to resolve them.

For example, the omission of an exploratory phase (i.e., participant observation, speaking the vernacular, rapport-building) can provide misleading results and show the ethnocentric nature of the stimuli being used (Leys, 2010). In the same vein, when they export Western procedures (e.g., forced-choice response formats based on lists of emotional terms or emotional antecedents scenarios) to indigenous societies, without any previous descriptive work, researchers merely implement a strategy destined to confirm their own preconceptions, and preclude discovery of indigenous modes of understanding. The selection of a list of emotion terms or stories relies not only on the vernacular, but also on the assumption that there is a clear one-to-one correspondence between English and the vernacular, as we indicated for the case of surprise in the Trobriand Islands. Likewise, it assumes that this one-to-one correspondence can be assessed in exclusively linguistic terms.

The Procrustean nature of much of previous uniformitarian research is exemplified by case of the *ekapunipuni matala* face (i.e., the gasping face) case, which illustrates how exporting and testing standard Western explanations can preclude understanding and explaining a phenomenon within the indigenous society’s terms. Only via the convergence of different sorts of evidence (e.g., Trobrianders’ explanations through conversations, interviews, material culture examples, etc.) could an apparent conundrum regarding the “non-recognition” of the standard “fear” display by Trobrianders have been resolved.

Our argument centers on the idea that anthropology should be re-instantiated within cognitive science as an equal partner with the other foundational sub-disciplines. Although there are multiple challenges that must be acknowledged (Beller et al., 2012), we claim that a multidisciplinary approach should be required to address relevant questions on cognitive science in indigenous societies.

## Author Contributions

All authors have made substantial, direct and intellectual contribution to the work, and have approved it for publication.

## Conflict of Interest Statement

The authors declare that the research was conducted in the absence of any commercial or financial relationships that could be construed as a potential conflict of interest.

## Footnotes

1 The category “Western” as we use it is admittedly a convenient fiction that considers the “West” to be uniform and unified. It is often taken to mean those societies that are roughly democratic, capitalistic, and permeated by American and/or European media.

2 The term *megwa* indicates the esoteric corpus of magic spells and practices that Trobrianders use in their daily lives to assist them with gardening, fishing, sailing, or courting. *Megwa* is a Kilivila word; Kilivila or *biga Boyowa* (“the word of Boyowa,” Boyowa being the local name for Kiriwina Island) is the vernacular. It belongs to the Austronesian family, Papuan tip cluster (Senft, 1986).

3 A thorough discussion of the “incommensurability of cultures” is beyond the scope of this paper, and we disagree with such positions *in extremis*. Nonetheless, we argue here that psychologists who go to the field with their theories in tow often compare the behavior of members of diverse societies while overlooking or oversimplifying critical contextual features that vitiate their comparisons. We further contend that the anthropological perspective can be extremely valuable for finding the “common ground” that allows cultural comparison without undermining description and context. Our approach, then, stresses the distillation of what is common ground by back-and-forth interplay rather than theoretical presumption.

4 We subscribe to Ingold’s (2014, p. 390) affirmation that the method of the anthropological discipline “lies in the observational engagement and perceptual attunement that allow the practitioner to follow what is going on, and in turn to respond to it.”

5 A version of this myth (labeled “SN 232 Baloma”) can be found in the Jerry W. Leach Trobriand Folklore Collection, part of the Jerry W. Leach Trobriand Papers and Sound Recordings at the National Anthropological Archives, Smithsonian Institution.

6 Other frequently used metaphors for describing emotion categories are *boluluki lopogu* (i.e., *my belly is full of resentment*), and *kaitigamala lopola* (i.e., delighted or happy belly).
